# Prevalence of Hashimoto Thyroiditis in Adults With Papillary Thyroid Cancer and Its Association With Cancer Recurrence and Outcomes

**DOI:** 10.1001/jamanetworkopen.2021.18526

**Published:** 2021-07-27

**Authors:** Siyuan Xu, Hui Huang, Jiaxin Qian, Yang Liu, Ying Huang, Xiaolei Wang, Shaoyan Liu, Zhengang Xu, Jie Liu

**Affiliations:** 1 Department of Head and Neck Surgical Oncology, National Cancer Center/National Clinical Research Center for Cancer/Cancer Hospital, Chinese Academy of Medical Sciences and Peking Union Medical College, Beijing, People’s Republic of China

## Abstract

**Importance:**

Hashimoto thyroiditis (HT) has been suggested to be associated with papillary thyroid cancer (PTC) development. However, its association with PTC progression remains unclear.

**Objective:**

To examine the association between HT and PTC presentation and outcomes.

**Design, Setting, and Participants:**

This retrospective cohort study included a review of patients aged 18 to 75 years who had pathologically confirmed PTC treated at a single center in China from January 1, 2001, to December 31, 2014. Data analysis was performed from November 1 to December 31, 2020.

**Exposures:**

Coexistent HT was defined according to evaluation of postoperative paraffin sections.

**Main Outcomes and Measures:**

The primary outcome was the association of HT with PTC-related mortality, assessed using Cox proportional hazards regression models. The secondary outcome was the association of HT with aggressive characteristics and structural recurrence of PTC, assessed using logistic regression and Cox proportional hazards regression with and without adjustment for related factors.

**Results:**

Of 9210 patients with PTC (mean [SD] age, 43.6 [12.0] years; 6872 [75%] women) included in the analysis, 1751 (19%) had HT. In the logistic regression model, HT was negatively associated with frequencies of primary tumor size of 4 cm or greater (adjusted odds ratio [aOR], 0.20; 95% CI, 0.12-0.33; *P* < .001), gross extrathyroidal extension (aOR, 0.44; 95% CI, 0.36-0.54; *P* < .001), extranodal extension (aOR, 0.66; 95% CI, 0.55-0.80; *P* < .001), and distant metastasis (aOR, 0.17; 95% CI, 0.04-0.71; *P* = .02). After a median follow-up of 85 months (range, 12-144 months), 131 PTC-related deaths were identified in the cohort; 2 patients who died had HT. Patients with HT had significantly superior outcomes compared with patients without HT in terms of unadjusted 10-year disease-specific survival (99.9% vs 96.6%; log-rank *P* < .001) and recurrence-free survival (92.0% vs 87.6%; log-rank *P* = .001). After adjusting for sex, age, primary tumor size, extrathyroidal extension, lymph node metastasis, distant metastasis, extent of surgery, and radioactive iodine ablation, HT was associated with decreased PTC-related mortality (hazard ratio [HR], 0.19; 95% CI, 0.05-0.76; *P* = .02). Stratified analysis showed that HT was associated with less frequent structural recurrence in patients with extrathyroidal extension (HR, 0.52; 95% CI, 0.38-0.71; *P* < .001; *P* = .002 for interaction) or after total thyroidectomy (HR, 0.50; 95% CI, 0.35-0.69; *P* < .001; *P* = .009 for interaction).

**Conclusions and Relevance:**

In this cohort study, patients with coexistent HT had less aggressive characteristics at presentation and better outcomes of PTC than did patients without HT. The findings suggest that autoimmune thyroiditis has a protective role in association with thyroid cancer.

## Key Points

**Question:**

Is Hashimoto thyroiditis associated with papillary thyroid cancer characteristics and outcomes?

**Findings:**

In this cohort study of 9210 patients with papillary thyroid cancer, 19% had Hashimoto thyroiditis. Patients with Hashimoto thyroiditis had less aggressive papillary thyroid cancer tumors, lower rates of tumor recurrence, and lower disease-related mortality compared with patients without Hashimoto thyroiditis.

**Meaning:**

The findings suggest that patients with Hashimoto thyroiditis have better outcomes of papillary thyroid cancer than do patients without Hashimoto thyroiditis.

## Teaser

his cohort study assesses the prevalence of and outcomes associated with Hashimoto thyroiditis among adults with papillary thyroid cancer.

## Introduction

Papillary thyroid cancer (PTC) accounts for more than 90% of all thyroid neoplasms.^1,2^ Most PTCs are associated with an indolent disease course and have a favorable prognosis even after low-intensity treatment. Therefore, disease management includes a wide range of options, from active surveillance to radical surgery and subsequent radioactive iodine (RAI) ablation.^3^ Consequently, the major clinical challenge is to stratify patients by risk of mortality or recurrence. At present, several clinicopathologic characteristics have been recognized as being associated with an unfavorable prognosis, including older age, large primary tumor size, extrathyroidal extension (ETE), lymph node metastasis (LNM), and distant metastasis.^3,4,5,6^ Patients with these risk factors thus require aggressive treatment, whereas low-intensity treatment may be sufficient for patients who do not have these risk factors.^3,4,7^

Hashimoto thyroiditis (HT) is the most common human autoimmune disease, and its prevalence has also increased in recent years.^8^ Epidemiologic studies have reported a mean coexistence rate between HT and PTC of approximately 23% (range, 5%-85%).^9,10^ Moreover, HT has been considered to be potentially associated with PTC development and progression since it was first described in 1955.^8^ Most studies suggest that HT may be a protective factor for PTC because it is associated with less invasive disease at presentation and a lower recurrence rate.^11,12,13^ This association is appealing and rational because the mechanism may be related to lymphocyte infiltration caused by HT and facilitating antitumor immunity.^14,15^ However, relevant evidence is lacking because no association was found in most multivariate analyses.^16,17^ These conflicting findings may, in part, be attributable to different diagnostic criteria used for HT, low PTC recurrence rates or PTC-related mortality rates, and insufficient study sample size. In addition, few results of the aforementioned studies regarding the association of HT with PTC-related mortality were found. We undertook the present study to assess the association of HT with the progression and prognosis of PTC by examining the association of aggressive characteristics at presentation, structural recurrence, and disease-related mortality of PTC with the presence of coexistent HT.

## Methods

This cohort study used retrospective data from consecutive patients aged 18 to 75 years who underwent thyroidectomy for PTC at the National Cancer Center, Chinese Academy of Medical Sciences, Cancer Hospital from January 1, 2001, to December 31, 2014. Data analysis was performed from November 1 to December 31, 2020. The study was approved by the ethics committee of the Cancer Hospital, Chinese Academy of Medical Sciences. Written informed consent was obtained at the time of surgery for general use of clinical information in future studies. This study followed the Strengthening the Reporting of Observational Studies in Epidemiology (STROBE) reporting guideline for observational studies.

Variables such as patient age, sex, preoperative serum autoantibody levels, tumor characteristics, and treatment modalities were obtained from the medical records. Patients with poorly differentiated PTC or other thyroid malignant neoplasms, a history of cancer or thyroid surgery, or insufficient follow-up data (≤12 months since initial treatment) were excluded from the analysis.

Coexistent HT was determined by postoperative sectioning and examination of paraffin-embedded thyroid tissue specimens; a positive result was defined as the presence of diffuse lymphocytic and plasma cell infiltrate, oxyphilic cells, formation of lymphoid follicles, and reactive germinal centers. The infiltrate must have been found in a normal region of the thyroid gland, distinct from the site of the PTC. A peritumoral inflammatory response was not considered to be evidence of HT. Serum antithyroglobulin and antithyroid peroxidase levels were measured within 30 days before surgery using the immune-electrochemiluminescence method, and results were considered positive when these levels exceeded 115 IU/mL and 34 IU/mL, respectively.

Primary tumor size, ETE, gross ETE, and LNM were defined by postoperative pathologic examination. Lymph node metastasis was considered to be absent if no lymph nodes were examined. Surgical procedures performed for primary tumors included lobectomy and total thyroidectomy; therapeutic neck dissection was performed in patients who had standard indications. Standard pathologic diagnoses were based on World Health Organization criteria.^18^ Postoperative treatments included conventional thyrotropin suppression at appropriate levels and RAI ablation. Survival outcomes were determined by medical records in combination with telephone follow-up. Local and regional recurrences were defined as structural disease as determined by either a cytologist or a pathologist. Distant metastasis was defined using computed tomography or emission computed tomography.

### Statistical Analysis

The study size was reflective of patients meeting eligibility criteria based on their histologic diagnosis and year of treatment. Clinicopathologic characteristics were compared across groups using the *t* test for continuous variables and the Pearson χ^2^ test for categorical variables. Logistic regression was performed to assess the association between HT and aggressive characteristics at presentation of PTC (primary tumor size ≥4 cm, ETE, gross ETE, LNM, lateral neck metastasis, extranodal extension, and distant metastasis) with and without adjustment for related factors. Kaplan-Meier survival curves and log-rank tests censoring patients at the time of last follow-up or 12 years and Cox proportional hazards regression analyses were used to compare PTC-related mortality and structural recurrence by presence or absence of coexistent HT. Cox proportional hazards regression models were adjusted for age and sex, and a second model was used to additionally adjust for other known prognostic factors (primary tumor size, ETE, LNM, distant metastasis, extent of surgery, and RAI ablation). Adjusted survival curves were created based on multivariate models and focused on the presence of HT. Stratified models were used to examine the association between HT and structural recurrences in subgroups (classified by age, sex, clinicopathologic factors, and different treatment strategies). Subgroup analyses were not adjusted for multiple comparisons. Interaction testing was also performed between subgroups. Significance levels were interpreted as 2-sided *P* < .05. All statistical analyses were performed using R, version 3.6.2 (R Foundation for Statistical Computing).

## Results

### Patient Characteristics

A total of 9210 patients (mean [SD] age, 43.6 [12.0] years; 6872 women [75%]) were included in the analysis. The overall prevalence of coexistent HT was 19% (1751 cases). Compared with patients without HT, patients with HT were more likely to be younger (mean [SD] age, 41.6 [11] years vs 44.1 [12] years) and female (92% vs 71%) and to have a smaller primary tumor size (mean [SD], 1.2 [0.7] cm vs 1.5 [1.2] cm), fewer metastatic nodes (mean [SD], 4 [8] vs 5 [9]), and a low lymph node ratio (mean [SD], 0.24 [0.22] vs 0.30 [0.28]). Patients with HT were less likely to have ETE (38% vs 43%), lateral neck metastasis (23% vs 28%), extranodal extension (16% vs 29%), more than 3 cm of nodal metastasis (1% vs 4%), or distant metastasis (0.1% vs 1.1%) (all *P* < .001) (Table 1).

**Table 1.** Demographic, Clinical, and Pathologic Characteristics According to Coexistence of HT

Characteristic	Patients (N = 9210)		*P* value
	HT absent (n = 7459)	HT present (n = 1751)	
Age, mean (SD), y	44.12 (12)	41.62 (11)	<.001
Sex, No. (%)			
Male	2196 (29)	142 (8)	<.001
Female	5263 (71)	1609 (92)	
Primary tumor size			
Mean (SD), cm	1.5 (1.2)	1.2 (0.7)	NA
<2 cm	5430 (73)	1440 (82)	<.001
2 to <4 cm	1588 (21)	294 (17)	
≥4 cm	441 (6)	17 (1)	
Extrathyroidal extension, No. (%)			
Absent	4231 (57)	1093(62)	<.001
Present	3228 (43)	658(38)	
Multifocality, No. (%)			
Absent	4769 (64)	1079 (62)	.07
Present	2690 (36)	672(38)	
Bilateral lesions, No. (%)			
Absent	5655 (76)	1311 (75)	.41
Present	1804 (24)	440 (25)	
Nodal status, No. (%)			
None	3713 (50)	874 (50)	<.001
N1a	1851 (22)	492 (27)	
N1b	1831 (28)	352 (23)	
Nodes removed, mean (SD), No.^a^	17.8 (23)	16.2 (24)	.65
Metastases, mean (SD), No.^a^	3.6 (7)	4.0 (7)	<.001
>3 cm Node metastasis, No. (%)^a^			
Absent	3590 (96)	875 (99)	<.001
Present	161 (4)	6 (1)	
Lymph node ratio, median (SD)^a^	0.30 (0.28)	0.24 (0.22)	<.001
Extranodal extension, No. (%)^a^			
Absent	2680 (71)	741 (84)	<.001
Present	1071 (29)	140 (16)	
Distant metastasis, No. (%)			
Absent	7379 (98.9)	1749 (99.9)	<.001
Present	80 (1.1)	2 (0.1)	
Stage, No. (%)			
I	6560 (88)	1620 (94)	<.001
II	653 (9)	105 (6)	
III	213 (3)	25 (1)	
IV	33 (0)	1 (0)	
Extent of surgery, No. (%)			
Lobectomy	4718 (63)	1059 (61)	.03
Total thyroidectomy	2741 (37)	692 (39)	
Radioactive iodine ablation			
Absent	5785 (77)	1339 (76)	.33
Present	1674 (23)	412 (24)	

Abbreviations: HT, Hashimoto thyroiditis; NA, not applicable.

^a^ Including patients with lymph node metastasis (n = 4526).

### Association Between HT and Aggressive Characteristics of PTC at Presentation

Logistic regression showed that HT was negatively associated with frequencies of primary tumor size of 4 cm or greater (odds ratio [OR], 0.16; 95% CI, 0.10-0.33; *P* < .001), ETE (OR, 0.79; 95% CI, 0.71-0.88; *P* < .001), gross ETE (OR, 0.36; 95% CI, 0.30-0.44; *P* < .001), lateral neck metastasis (OR, 0.74; 95% CI, 0.66-0.84; *P* < .001), extranodal extension (OR, 0.52; 95% CI, 0.43-0.62; *P* < .001), and distant metastasis (OR, 0.11; 95% CI, 0.03-0.43; *P* = .002) (Table 2). After adjusting for age and sex, HT was negatively associated with primary tumor size of 4 cm or greater (adjusted OR [aOR], 0.20; 95% CI, 0.12-0.33; *P* < .001), and after additionally adjusting for primary tumor size, HT was negatively associated with gross ETE (aOR, 0.44; 95% CI, 0.36-0.54; *P* < .001), extranodal extension (aOR, 0.66; 95% CI, 0.55-0.80; *P* < .001), and distant metastasis (aOR, 0.17; 95% CI, 0.04-0.71; *P* = .02) (Table 2).

**Table 2.** Aggressive Characteristics at Presentation of Papillary Thyroid Cancer and ORs for Coexistent vs Absent Hashimoto Thyroiditis by Logistic Regression

Aggressive characteristic	Unadjusted OR (95% CI)	*P* value	Adjusted OR (95% CI)^a^	*P* value
Primary tumor size ≥4 cm	0.16 (0.10-0.33)	<.001	0.20 (0.12-0.33)	<.001
Extrathyroidal extension	0.79 (0.71-0.88)	<.001	0.90 (0.81-1.01)	.08
Gross extrathyroidal extension	0.36 (0.30-0.44)	<.001	0.44 (0.36-0.54)	<.001
Lymph node metastasis	1.00 (0.90-1.11)	.98	NA	
Lateral neck metastasis^b^	0.74 (0.66-0.84)	<.001	0.93 (0.82-1.07)	.31
Extranodal extension^b^	0.52 (0.43-0.62)	<.001	0.66 (0.55-0.80)	<.001
Distant metastasis	0.11 (0.03-0.43)	.002	0.17 (0.04-0.71)	.02

Abbreviations: NA, not applicable; OR, odds ratio.

^a^ Odds ratio adjusted for sex, age, and primary tumor size except for primary tumor size ≥4 cm, which was adjusted for sex and age.

^b^ Including patients with lymph node metastasis (n = 4526).

### Association Between HT and PTC-Related Mortality

**Figure 1.**
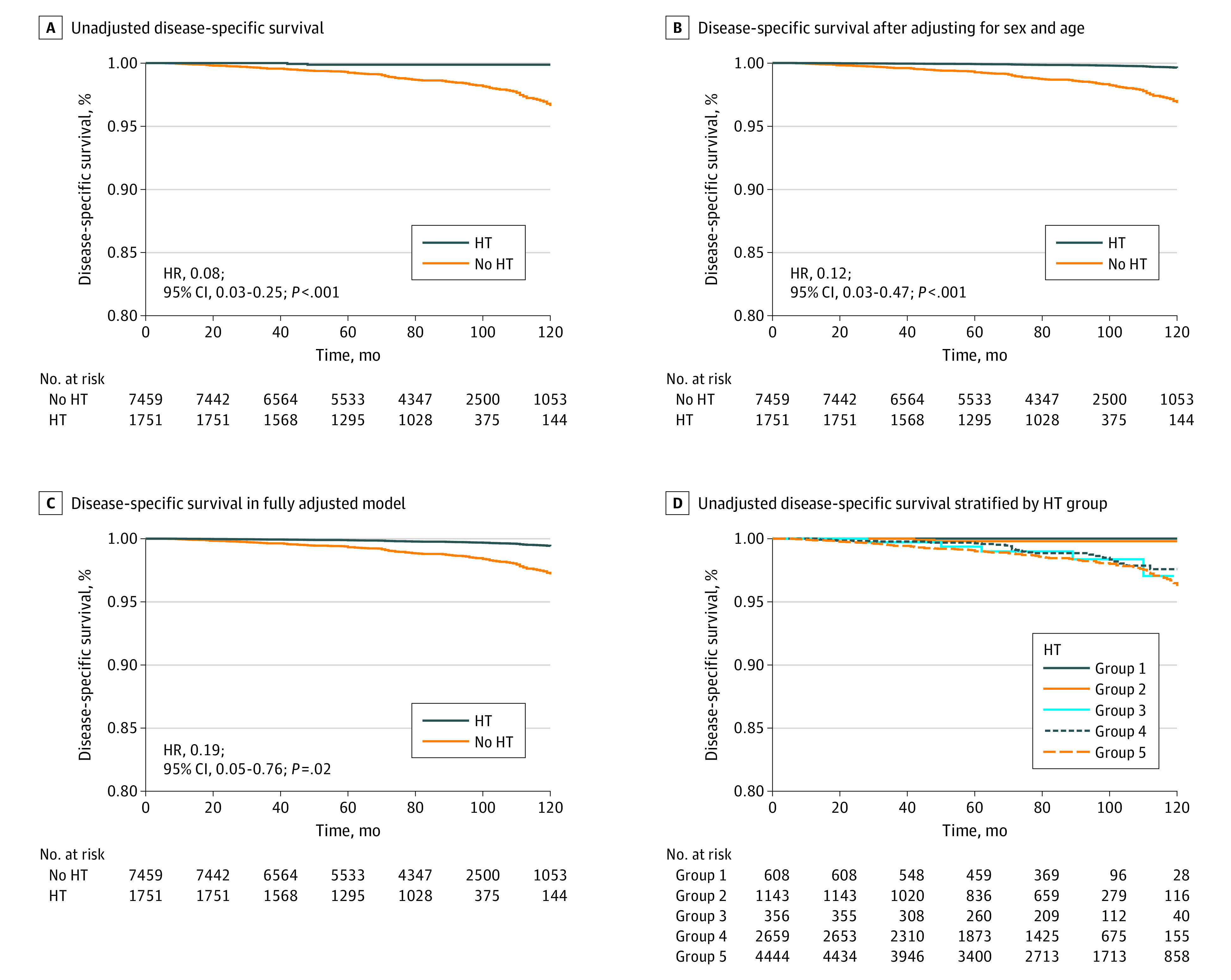
Disease-Specific Survival Among Patients in the Study Cohort C, Model was adjusted for sex, age, primary tumor size, extrathyroidal extension, lymph node metastasis, distant metastasis, extent of surgery, and radioactive iodine ablation. D, Patients were stratified by postoperative pathologic diagnosis of Hashimoto thyroiditis (HT) and serum antibody status: group 1, pathologically confirmed HT and positive serum antibody status; group 2, pathologically confirmed HT alone and unknown antibody status; group 3, positive antibody status and no pathologic evidence of HT; group 4, normal antibody levels and no pathologic evidence of HT; group 5, unknown antibody levels and no pathologic evidence of HT.

The median follow-up period for the whole cohort was 85 months (range, 12-144 months), and no significant difference in follow-up time was observed between patients with and without HT (median, 84 months [range, 30-144 months] vs 85 months [range, 12-144 months]; *P* = .08). Of 131 patients who died of PTC-related causes, 2 had HT. According to the Kaplan-Meier curves, unadjusted 10-year disease-specific survival rates among patients with HT (99.9%) were significantly higher than those among patients without HT (96.6%) (log-rank *P* < .001) (Figure 1A).

According to Cox proportional hazards regression models, HT was associated with decreased PTC-related mortality after adjusting for sex and age (hazard ratio [HR], 0.12; 95% CI, 0.03-0.48; *P* = .003) (Figure 1B) and after adjusting for sex, age, primary tumor size, ETE, LNM, distant metastasis, extent of surgery, and RAI ablation (HR, 0.19; 95% CI, 0.05-0.76; *P* = .02) (Figure 1C). In stratified analyses, HT was associated with decreased PTC-related mortality in a subgroup of patients 45 years or older (HR, 0.10; 95% CI, 0.03-0.42; *P* < .001) and in a subgroup of patients who underwent total thyroidectomy (HR, 0.12; 95% CI, 0.03-0.49; *P* < .001) (eFigure in the Supplement). Hazard ratios for subgroups of patients younger than 45 years and those who had a lobectomy were not available because no PTC-related mortality was observed among the patients with HT.

Preoperative serum thyroid autoantibody (antithyroglobulin and antithyroid peroxidase) levels were available for 4641 patients. Further stratification was performed as follows: 608 patients had both pathologically confirmed HT and positive serum antibody status (antithyroglobulin or antithyroid peroxidase) (group 1), 1143 had pathologically confirmed HT alone (1018 with negative antibody status and 125 with unknown antibody status) (group 2), 356 patients had positive antibody status (antithyroglobulin and antithyroid peroxidase) but no pathologic evidence of HT (group 3), 2569 had normal antibody levels and no pathologic evidence of HT (group 4), and 4444 had unknown antibody levels and no pathologic evidence of HT (group 5). According to the Kaplan-Meier curve, patients with pathologically confirmed HT (groups 1 and 2) had lower PTC-related mortality rates than did patients with no pathologic evidence of HT regardless of antibody status (10-year disease-specific survival: group 1, 100%; group 2, 99.8%; group 3, 97.0%; group 4, 97.6%; and group 5, 96.3%) (Figure 1D).

### Association Between HT and Structural Recurrence

**Figure 2.**
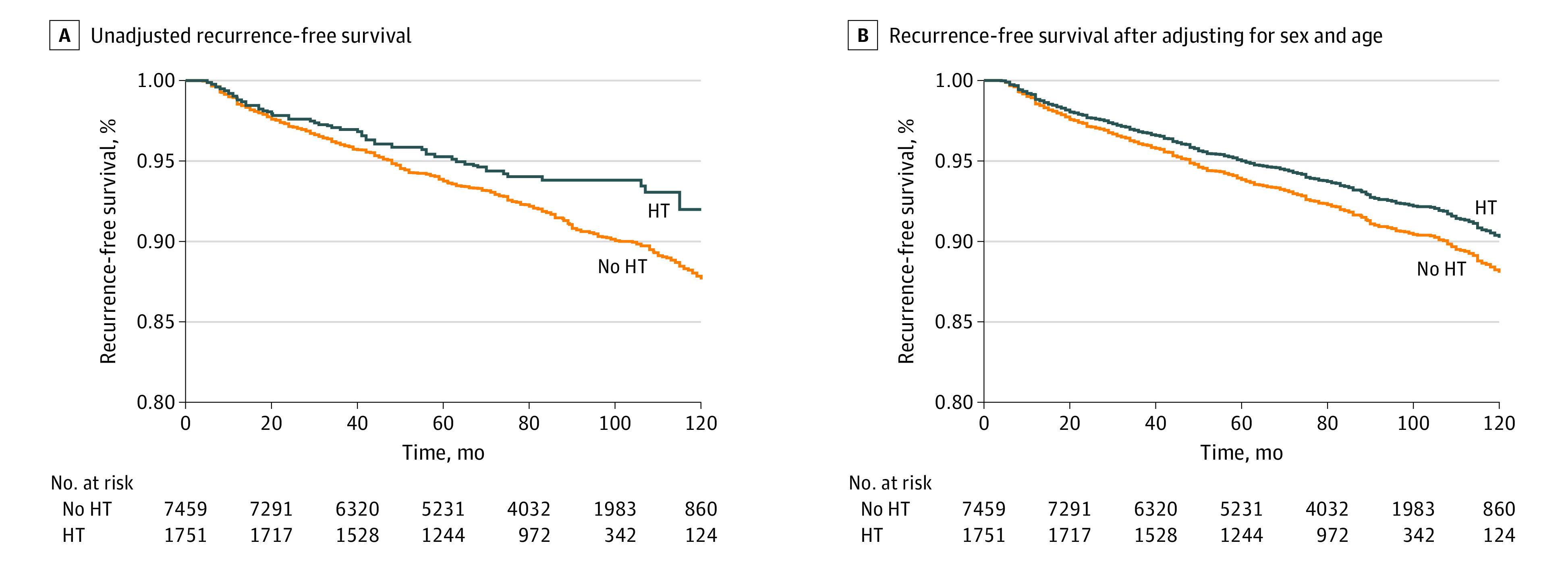
Recurrence-Free Survival Among Patients in the Study Cohort HT indicates Hashimoto thyroiditis.

During follow-up, 100 and 633 structural recurrences of PTC were identified in patients with and without HT, respectively. Hashimoto thyroiditis was associated with greater 10-year recurrence-free survival (92.0% vs 87.6%; log-rank *P* = .001) (Figure 2A); there was no association after adjusting for sex and age (HR, 0.81; 95% CI, 0.65-1.00; *P* = .052) (Figure 2B). Stratified analysis showed that HT was negatively associated with structural recurrence in patients younger than 45 years, those 45 years or older, male patients, those with a primary tumor size of 2 cm or greater, those with ETE and LNM, those who underwent total thyroidectomy, and those with and without history of RAI ablation. Interaction testing showed that HT was associated with less structural recurrence in patients with ETE and in those who had undergone total thyroidectomy (Table 3).

**Table 3.** Stratified Analysis of Unadjusted Hazard Ratios of Structural Recurrence for Coexistent vs Absent Hashimoto Thyroiditis in Cox Proportional Hazards Regression Models

Stratification	Hazard ratio (95% CI)	*P* value	*P* value for interaction
Age, y			
<45	0.76 (0.58-0.98)	.04	.29
≥45	0.59 (0.41-0.84)	<.001	
Sex			
Female	0.88 (0.70-1.11)	.27	.07
Male	0.47 (0.23-0.94)	.03	
Primary tumor size			
<2 cm	0.95 (0.73-1.25)	.72	.06
≥2 cm	0.61 (0.43-0.86)	.005	
Extrathyroidal extension			
Absent	1.08 (0.81-1.44)	.61	.002
Present	0.52 (0.38-0.71)	<.001	
Lymph node metastasis			
Absent	0.73 (0.48-1.12)	.15	.82
Present	0.68 (0.53-0.87)	.002	
Extent			
Lobectomy	0.88 (0.67-1.15)	.35	.009
Total thyroidectomy	0.50 (0.35-0.69)	<.001	
Radioactive iodine administration			
Absent	0.74 (0.59-0.93)	.01	.55
Present	0.59 (0.35-0.99)	.046	

## Discussion

In this cohort study, we identified a significant negative association of coexistent HT with PTC-related mortality in conjunction with both aggressiveness of the present tumor and structural recurrence. These findings suggest a protective role of HT against PTC progression. We also observed that the association was more significant in patients with factors (ie, ETE) associated with structural recurrence, which may be the reason that PTC-related mortality was rare (2 deaths) among patients with coexistent HT.

Coexistent HT has been reported to be significantly associated with the less aggressive clinicopathologic characteristics of PTC,^17,19,20^ but its association with prognosis remains controversial. Although several studies revealed longer recurrence-free survival among patients with coexistent HT, few multivariate analyses have confirmed its significance as an independent factor associated with decreasing recurrence.^21^ Moreover, the association between HT and PTC-related mortality has not been established. This inconsistency may be attributable to the low mortality rate associated with PTC and the protective effect of HT not being independent of tumor characteristics. Our study revealed a significant association between HT and PTC-related mortality after adjustment for multiple factors, and stratified analysis showed that the association of HT with less recurrence may have been stronger among patients with certain risk factors, such as ETE. Interactions were significant between the subgroups based on primary tumor size, which was another risk factor for recurrence. These findings suggest that HT may restrict tumor progression through a certain mechanism and were consistent with the survival outcomes. In this study, PTC-related mortality among patients with HT was rare (2 of 1751); similar findings were reported in previous studies.^16,22^ In a study by Kashima et al,^16^ 2 of 281 deaths from cancer were reported among patients who had coexistent PTC and HT. Loh et al^22^ reviewed 128 patients with coexisting PTC and HT, and after a mean follow-up period of approximately 11 years, only 1 death from cancer (0.8%) was detected.

The diagnostic criteria for HT may also have influenced these results. In this study, the proportion of coexistent HT in patients with PTC was 19%, which is similar to that reported in the literature, and the study period (from January 1, 2001, to December 31, 2014) was selected to balance the pathologic diagnosis of HT and follow-up time. In addition, although it is common to make a pathologic diagnosis when evaluating patients with PTC for HT, findings from some studies have suggested that serum antibodies alone may be associated with the prognosis of PTC.^23,24,25^ In this study, we also collected serum autoantibody data in conjunction with pathologic diagnoses. This was to strengthen the diagnostic reliability of a diagnosis of HT and to examine its predictive value. Serum antibody status had a weaker association with prognosis than did pathologic diagnosis, which suggests that pathologic changes may have a stronger association with PTC progression.

### Limitations

This study has limitations. First, in the study cohort, the proportion of patients who underwent lobectomy was greater than that in other studies,^7^ and RAI ablation was performed less often in the study cohort, which could have affected the outcomes. According to findings from previous studies,^26,27^ this conservative approach likely did not compromise long-term survival in the present study. Stratification analysis of both mortality and recurrence showed the protective effect of HT in the total thyroidectomy subgroup, and results were similar between patients who had and had not undergone RAI ablation. Second, because of the small number of PTC-related deaths, especially in patients with HT (2 deaths), it was impossible to perform a comprehensive stratified analysis. However, owing to the large number of cases of coexistent HT, both the unadjusted and adjusted disease-specific survival showed significant differences between patients with and without HT, and stratified analysis on recurrence showed coincident results in most subgroups with advanced disease.

## Conclusions

In this cohort study, coexistent HT was associated with a lower prevalence of aggressive characteristics of PTC and a better prognosis among patients with PTC. The findings suggest that autoimmune thyroiditis has a protective role in association with thyroid cancer.

## Notes

Supplement. **eFigure.** Stratified Analyses for Patients Aged 45 Years and Older and Patients Who Underwent Total Thyroidectomy
