# Supplementary material for: Prevalence of Hashimoto Thyroiditis in Adults With Papillary Thyroid Cancer and Its Association With Cancer Recurrence and Outcomes
Source: JAMA Netw Open. 2021 Jul 27;4(7):e2118526. doi: 10.1001/jamanetworkopen.2021.18526 (PMC8317012; doi:10.1001/jamanetworkopen.2021.18526)
Supplement: Supplement. — eFigure. Stratified Analyses for Patients Aged 45 Years and Older and Patients Who Underwent Total Thyroidectomy [file jamanetwopen-e2118526-s001.pdf]

Supplementary Online Content

Xu S, Huang H, Qian J, et al. Prevalence of Hashimoto thyroiditis in adults with papillary thyroid cancer and its association with cancer recurrence and outcomes. *JAMA Netw Open*. 2021;4(7):e2118526. doi:10.1001/jamanetworkopen.2021.18526

eFigure. Stratified Analyses for Patients Aged 45 Years and Older and Patients Who Underwent Total Thyroidectomy

This supplementary material has been provided by the authors to give readers additional information about their work.

© 2021 Xu S et al. *JAMA Network Open*.

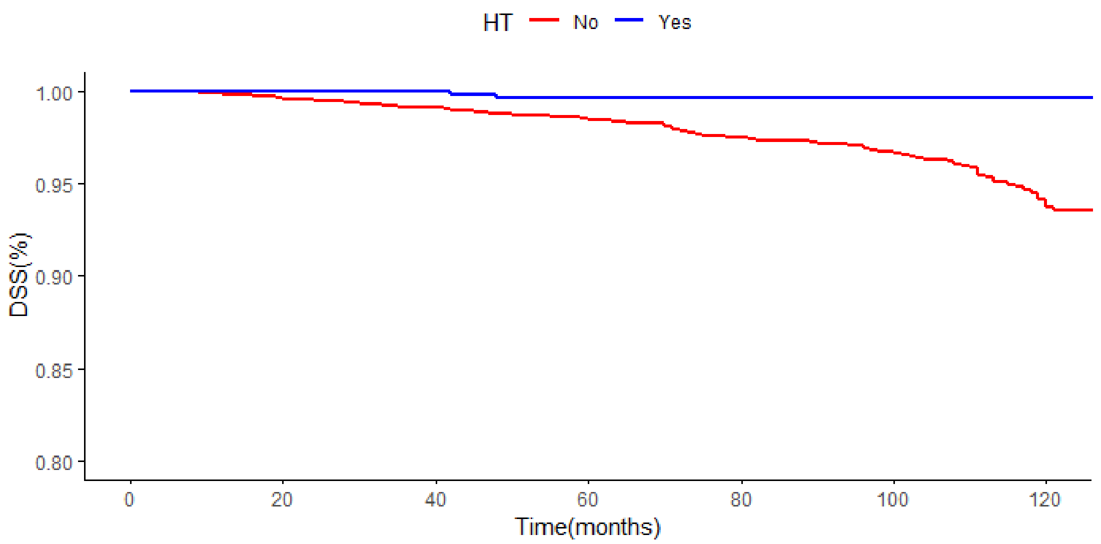

Number at risk

No	3600	3583	3128	2616	2008	1155	492
Yes	688	688	617	517	398	138	52

A

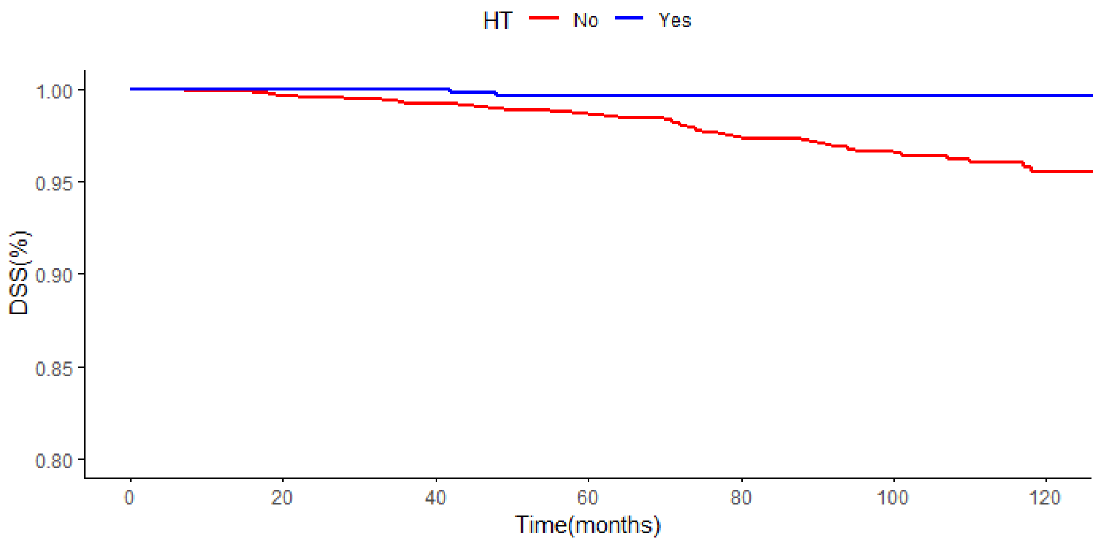

Number at risk

No	2741	2728	2384	1970	1482	749	304
Yes	692	692	610	488	383	100	35

B

eFigure 1 A, Unadjusted disease-specific survival of patients with and without HT in patients age≥45 years (P<0.01). B, Unadjusted disease-specific survival of patients with and without HT in patients who underwent total thyroidectomy (P<0.01).

© 2021 Xu S et al. *JAMA Network Open*.
